# Pattern of care for re-irradiation in locally recurrent rectal cancer: a national survey on behalf of the AIRO gastrointestinal tumors study group

**DOI:** 10.1007/s11547-023-01652-3

**Published:** 2023-06-26

**Authors:** Giovanna Mantello, Elena Galofaro, Luciana Caravatta, Clelia Di Carlo, Sabrina Montrone, Donatella Arpa, Giuditta Chiloiro, Antonino De Paoli, Vittorio Donato, Maria Antonietta Gambacorta, Domenico Genovesi, Marco Lupattelli, Gabriella Macchia, Giampaolo Montesi, Rita Marina Niespolo, Elisa Palazzari, Antonio Pontoriero, Melissa Scricciolo, Francesca Valvo, Pierfrancesco Franco

**Affiliations:** 1 Radiotherapy Department, Azienda Ospedaliero Universitaria delle Marche, Via Conca 71, 60126 Ancona, Italy; 2 Department of Radiation Oncology, SS Annunziata Hospital, Chieti, Italy; 3 Department of Radiotherapy, Pisa University, Pisa, Italy; 4 IRCCS Istituto Scientifico Romagnolo per lo Studio dei Tumori (IRST) “Dino Amadori”, Radiotherapy Unit, Meldola, Italy; 5 Departments of Radiation Oncology, Fondazione Policlinico Universitario A. Gemelli IRCCS, Rome, Italy; 6 Division of Radiation Oncology, Centro di Riferimento Oncologico di Aviano (CRO) IRCCS, Aviano, Italy; 7 Radiation Oncology Division, Oncology and Speciality Medicine Department, San Camillo-Forlanini Hospital, Rome, Italy; 8 Radiation Oncology Section, University of Perugia and Perugia General Hospital, Perugia, Italy; 9 Radiotherapy Unit, Gemelli Molise Hospital, Catholic University of Sacred Heart, Campobasso, Italy; 10 Radiotherapy Unit, ULSS5, Rovigo, Italy; 11 Radiotherapy Unit, Azienda Ospedaliera San Gerardo, Monza, Italy; 12 Radiation Oncology Unit, Department of Biomedical, Dental Science and Morphological and Functional Images, University of Messina, Messina, Italy; 13 UOC di Radioterapia Oncologica Mestre, Ospedale dell’Angelo, Venice, Mestre, Italy; 14 Fondazione CNAO, National Center of Oncological Hadrontherapy, Pavia, Italy; 15 Department of Translational Medicine, Department of Radiation Oncology, Maggiore Della Carità University Hospital, University of Eastern Piedmont, Novara, Italy

**Keywords:** Recurrence, Rectal cancer, Re-irradiation, AIRO gastrointestinal tumors study group, National survey

## Abstract

**Purpose:**

Radical resection (R0) represents the best curative treatment for local recurrence (LR) rectal cancer. Re-irradiation (re-RT) can increase the rate of R0 resection. Currently, there is a lack of guidelines on Re-RT for LR rectal cancer. The Italian Association of Radiation and clinical oncology for gastrointestinal tumors (AIRO-GI) study group released a national survey to investigate the current clinical practice of external beam radiation therapy in these patients.

**Material and methods:**

In February 2021, the survey was designed and distributed to members of the GI working group. The questionnaire consisted of 40 questions regarding center characteristics, clinical indications, doses, and treatment techniques of re-RT for LR rectal cancer.

**Results:**

A total of 37 questionnaires were collected. Re-RT was reported as an option for neoadjuvant treatment in resectable and unresectable disease by 55% and 75% of respondents, respectively. Long-course treatment with 30–40 Gy (1.8–2 Gy/die, 1.2 Gy bid) and hypofractionated regimen of 30–35 Gy in 5 fractions were used in most centers. A total dose of 90–100 Gy as EqD2 dose (*α*/*β* = 5 Gy) was delivered by 46% of the respondents considering the previous treatment. Modern conformal techniques and daily image-guided radiation therapy protocols were used in 94% of centers.

**Conclusion:**

Our survey showed that re-RT treatment is performed with advanced technology that allow a good management of LR rectal cancer. Significant variations were observed in terms of dose and fractionation, highlighting the need for a consensus on a common treatment strategy that could be validated in prospective studies.

## Introduction

Rectal cancer represents one of the most common malignancies in Italy, with an age-standardized rate of 1.73 per 100.000 person-years worldwide. Despite multimodality approaches with preoperative radiotherapy (RT), chemotherapy (CT) and surgery (total mesorectal excision, TME) have led to a decrease in the incidence in LR [1–8], the rate of this event can still reach 10% [9–13], with 75% of cases occurring within 2 years from diagnosis [14, 15]. This event remains a severe clinical condition, with a relevant impact on the patient’s health-related quality of life (HR-QoL) due to debilitating symptoms such as pelvic pain, incontinence, bowel obstruction, fistula and/or bleeding and the management of this condition remains a challenge in daily clinical practice.

Treatment options include radical surgery, which is strongly related with long-term survival. Indeed, 5-year overall survival rates of 48–58% have been reported for tumor-free margins, whereas resection with close or positive margins (R1/2) results in 5-year survival rates of 10–18% [16–18]. Re-RT may increase the rate of radical resection (*R*0) and may also provide symptom palliation for inoperable tumors [19–21]. The potential of severe late toxicity has been the most major barrier to re-RT, limiting this option to a highly selected category of patients.

About 20 years ago, the Italian study group for therapies of rectal malignancies (STORM) conducted a multicentric phase II study to evaluate the efficacy and treatment-related toxicity of preoperative hyperfractionated chemoradiation for LR rectal cancer in patients previously irradiated to the pelvis. Conformal three-dimensional (3D) RT was planned and delivered on extended treatment volumes (gross tumor volume (GTV) plus 4 cm of radial margin), with a boost dose on a smaller volume (GTV plus 2 cm of radial margin) [22].

In the era of more advanced radiotherapy techniques, including intensity-modulated radiation therapy (IMRT), volumetric modulated arc therapy (VMAT), stereotactic body radiotherapy (SBRT) and IGRT allowing organs at risk (OARs) sparing with decreased toxicities, new RT schedules have been evaluated and a new scenario of re-RT strategy could be offered, although no shared recommendations on indications, doses and treatment techniques are currently available.

Based on these considerations, on behalf of the AIRO-GI, we conducted a national survey with the aim of investigating current clinical practice in the re-RT of recurrent rectal cancer with external beam radiotherapy (EBRT).

## Materials and methods

The project was developed within the AIRO-GI, whose Directive Council acted as a steering committee, in collaboration with the re-irradiation study group of the Italian Association of radiotherapy and clinical oncology (AIRO), with the purpose of assessing the current therapeutic practice in the scenario of locally recurrent rectal cancer re-RT. An external panel of radiation oncologists with a specific expertise in the management of rectal cancer provided suggestions and comments, creating and approving the survey in February 2021. The survey was compliant with the CHERRIES guidelines for reporting results of internet e-surveys [23].

In March 2021, the survey was carried out using Survey Monkey (www.surveymonkey.com), with an automatic method for capturing responses, tested and addressed to all members of the GI working group. Participants were informed about the survey at a meeting of the AIRO-GI and were invited to participate voluntarily via email. No personal information was collected. Professional information was stored within the Survey Monkey platform and protected from unauthorized access, as compliant with the platform regulatory. Demographics and professional information useful for stratification were collected. The project was approved by the Scientific Council and the Board of Directors of AIRO. No explicit informed consent was requested. No incentive was offered. The study allowed just one radiation oncologist per center to participate, with the restriction that they be experts in the treatment of gastrointestinal malignancies in general, and rectal cancer in particular. The password-protected questionnaire could only be completed once, and answers could not be changed. The questionnaire consisted of 40 multiple choice or open-ended questions regarding center characteristics, main indications, prescription dose, dose constraints, radiotherapy technique, and diagnostic exams.

The returned questionnaires were collected and analyzed at our Institute, Azienda Ospedaliero Universitaria delle Marche of Ancona, by an electronic database.

## Results

Among the 183 RT departments documented in Italy by AIRO (as per 2020), a total of 37 (20%) questionnaires were collected and considered for the current analysis.

The geographical distribution of the participating centers was 57%, 32%, and 11% in North, Central and South Italy, respectively, and in the 57% of cases, they were public hospitals. Over 50% of the participants had more than 10 years of experience and in about 80% of centers the radiation oncologist was dedicated to lower GI malignancies. Rectal cancer patients were treated by a multidisciplinary team in 97% of the locations.

The 67% of the centers treated more than 20 patients/year affected by rectal cancer with neoadjuvant intent; while, in the case of recurrent rectal cancer, 67% of adherent centers treated less than 5 cases/year, the 22% treated 5–10 patients per year and 11% treated more than 10 cases.

### Diagnosis and staging

We asked survey respondents which diagnostic tests were required if LR was suspected, giving them the opportunity to select several options. The most frequently prescribed examination for diagnosis and staging was magnetic resonance imaging of the pelvis. Computed tomography (CT) with contrast enhancement of the abdomen was prescribed in 97% of cases, thoracic CT in 88% and 9% of the cases “always” and “in selected cases”, respectively, whereas eco-endoscopy was prescribed “always” or “in selected cases” in 43% and 46% of cases, respectively. In addition, 40% and 57% of centers prescribed PET/TC “always” or “in selected cases”, respectively.

Recto-sigmoidoscopy and biopsy confirmation were always performed in 88% and 76% of centers, respectively, while they were required in 12% and 19% in selected cases. Carcinoembryonic antigen (CEA) determination in blood was always required by 94% of centers.

### Classification systems for locally recurrent rectal cancer

Criteria for classifying LR were examined. Respondents could select several options for classifying LR. Thirty-six (97%) respondents answered this question. In 30 of the 36 responding centers, the location of the LR is considered (central perianastomotic, vs. anterior, vs. lateral, vs. posterior). In addition, some centers also consider the size of the recurrence (16 centers), the infiltration of pelvic organ/pelvic wall (26 centers), and the clinical condition of the patient (11 centers).

### Re-RT role and Re-RT suitable time

We investigated the role of re-RT in patients with recurrent rectal cancer. In resectable LRs, 55% of centers emphasize that radiotherapy can play a preoperative role; in unresectable disease presentations, 75% of centers find a role for preoperative radiotherapy; operated LRs are considered treatable with adjuvant radiotherapy in 25% of centers. All centers consider a role for radiotherapy in palliation.

The appropriate time interval for re-RT was considered to be at least 6 months in 53% and 12 months in 44% of the centers. Only 3% of centers do not consider the time interval between first treatment and re-RT.

### Simulation

Most centers performed the CT simulation without contrast enhancement. Thirteen (35%) centers, always or in selected cases, administered iodinated contrast enhancement, while 33% of the centers performed MRI or PET-CT for planning procedures.

### Target volumes

For target delineation, MRI was used by 83% of responders, while PET-CT was required in the 28% and 72% always and in selected cases, respectively.

Twenty-nine (78%) centers answered the question regarding the margin to be added to the GTV to create the CTV: 4 centers (14%) adding no margin from the GTV to the CTV, the GTV–CTV margin was 0.5 cm, 1 cm, or more than 1 cm in 7%, 48%, and 31%, respectively.

Thirty-one (84%) centers answered the question on the margin to be added to the CTV to create the PTV. The CTV–PTV margin was 0.5 cm in 74%, 0.8 cm in 6%, 1 cm in 10%, and the rest of the respondents added 0.3 cm, 0.7 cm, or 4 cm. The margin was isotropic in 86% of the centers.

### Dose prescription and fractionations

We asked centers participating in the survey to indicate which fractionation schemes they use in clinical practice. Participants could select multiple options. Thirty-five centers responded with the option of standard/hyperfractionation. A total dose of 30–40 Gy, 40–50 Gy, and < 30 Gy is administered in 66% (23/35), 29% (10/35), and 17% (6/35) of centers, respectively. Thirty-four centers answered the question about the daily dose used, with several options to choose from. A daily dose/fraction of 1.8 and 2 Gy was administered in 51% and 45% of centers, respectively, while a hyperfractionated regimen of 1.2 and 1.5 Gy twice a day was chosen in 36% and 15% of responders, respectively.

For hypofractionated treatment, 34 centers answered the question. There were several options to choose from. A 5-fraction regimen was used in most of the centers, and 6 Gy/fraction, 7 Gy/fraction, and 8 Gy/fraction were used in 62%, 32%, and 6% of centers, respectively. Only 2 centers used a 3-fraction regimen (10 Gy/die).

The equivalent dose in 2-Gy fractions (EQD2) considered for RT was explored. Several options were available for selection. An alfa/beta value (*α*/*β*) of 5 Gy was used to calculate EQD2 in 61% of centers, and a *α*/*β* value of 10 Gy was used in 59% of centers.

**Fig. 1:**
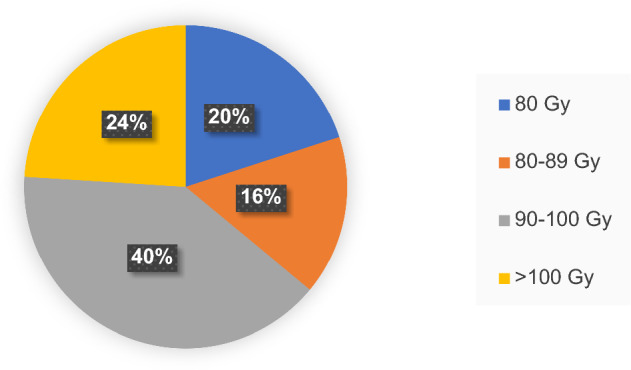
EQD2 sum of re-RT

The total EQD2 dose considering initial treatment RT and re-treatment RT is shown in Figure (Fig. 1).

Maximal total dose considered in planning optimization for OARs and the bibliographic reference were also investigated. Only 4 radiation oncologists answered this question. Two of them reported maximal total dose constraints according to Das et al. [24] and Abusaris et al. [25]. The other two declared that the clinical cases are individually evaluated according to the ALARA (as low as reasonably achievable) principle.

### Radiotherapy technique of re-irradiation

We allowed survey participants to choose among several options regarding the techniques used by the different centers. Of the 35 centers that responded, the majority use IMRT/VMAT (33 of 35 centers that responded) or SBRT (25 of 35 centers) techniques for re-RT. Only a few centers (3 of 35 centers) considered the possibility of using 3DCRT techniques. Five centers also indicated the possibility of using IORT or perioperative brachytherapy at their center, five centers indicated the possibility of using protons or carbon ions, and one center indicated the possibility of using brachytherapy.

All centers used protocols of daily IGRT (CBCT or MVCT) both for conventional and hypofractionated schedule, which allowed irradiation of smaller volumes. Real-time fiducial tracking was used in selected cases by 3 centers.

### Concomitant chemotherapy

Concomitant chemotherapy was prescribed to all patients or only in selected cases after multidisciplinary discussion in the 48% and 19% of centers, respectively. The remaining did not administer concomitant chemotherapy with re-RT.

### Evaluation of treatment response

The timing of treatment response evaluation was 7–8 weeks in the 58% of centers, over 8 weeks in the 17% and 4–6 weeks in the 23%. Pelvic MRI, abdomen/thorax CT, recto-sigmoidoscopy and CEA levels were mostly prescribed exams. RECIST dimension parameters were used by 91% of centers as criteria for evaluating response to treatment. In addition, 79% of centers also used pelvic organ infiltration/contact to assess response to treatment.

## Discussion

Rectal cancer is a common malignancy and about half of patients have locally advanced disease at diagnosis. Although neoadjuvant chemoradiation therapy followed by radical surgery can lead to good clinical outcomes, 10–20% of patients with rectal cancer develop LR [1–8]. Radical surgery represents the best treatment option for LR in terms of long-term survival. Unfortunately, a marginal-free surgery (*R*0) can only be performed in 20–30% of LR rectal cancer patients due to comorbidities or unresectable disease, which depends on the site, size and near tissues infiltration. Therefore, most patients require extensive surgery with high morbidity to achieve cure [18, 19].

There is still no clarity on the best management for a patient with recurrence of rectal cancer. The goal of this survey was to highlight the main issues and to understand where more homogeneity is needed.

First, it is clear how the patient should be classified uniformly. Over the years, many classification methods have been proposed [26–28]. All of these classifications of recurrence prioritize a clear assessment of the disease in terms of resectability [29]. In an attempt to shed light, a recent review proposed by Rokan and colleagues highlights the various imaging systems used to classify LR rectal cancer and some of the prognostic indicators of survival and oncologic clearance based on these systems [30]. In our survey, respondents could select several options for the classification of LR. In 83% of center, the pattern of pelvic invasion is taken into account (central perianastomotic, vs. anterior, vs. lateral, vs. posterior). In addition, some centers also consider size of the recurrence, infiltration into the pelvic organs and the clinical condition of the patients. Given that the use of each classification system may depend on institutional preferences, it seems clear that a common classification could lead to clear therapeutic management of these patients by allowing common decisions to be made in all centers about resectability and the appropriate timing of surgery, radiotherapy, and chemotherapy.

Our survey also showed that different prescription regimes are used in patients eligible for re-RT. We gave the possibility to choose multiple responses regarding treatment regimens. The most common fractionation chosen was 30–40 Gy with a daily dose/fraction of 1.8–2 Gy. About half of the centers considered concomitant chemotherapy after multidisciplinary discussion.

To date, the preferred schedule for RT fractionation has not been standardized. Older studies have shown that hyperfractionation RT is feasible with or without concurrent chemotherapy and is the preferred option in a curative setting. A hyperfractionation regimen was studied in 59 patients in a multicenter phase II trial promoted by the study group for therapies of rectal malignancies (STORM) in the late 1990s. A conformal 3DRT was planned and a total dose of 30 Gy, 1.2 Gy twice daily, was administered 5 days a week to a fairly large treatment volume (GTV plus 4 cm radial margin). A boost dose of 10.8 Gy was administered to a smaller treatment volume (GTV plus 2 cm radial margin). The incidence of grade 3 acute gastrointestinal toxicity was only 5% with an acceptable incidence of late complications (2 skin fibrosis, 2 impotence, 2 urinary complications requiring nephrostomy, and 1 small bowel fistula requiring surgical diversion). The median overall survival was 42 months, and the actuarial 5-year survival rate was 39% (67% in *R*0-resected patients and 22% in patients treated without surgery or with subtotal tumor removal) [22].

2-day hyperfractionation is considered in only a small proportion of centers. The study STORM was performed with 3D techniques. Given the new technologies that allow highly conformal targeting and daily imaging of the lesion and organs at risk, we could confidently push for higher doses of at least 40–50 Gy with daily fractionation of 1.8–2 Gy.

Hypofractionation is a therapeutic option for radiation oncologists who responded to the survey with the most widely used regimen of 5 fractions in ablative RT, and the most used daily fractionation was 6 Gy. Given the lack of phase III studies on hypofractionation in patients undergoing re-irradiation for rectal cancer recurrence, there is still perplexity about using a higher daily fractionation dose for fear of short- and long-term side effects. A good dosimetric evaluation of the previous treatment and further studies are needed to find the right fractionation for this patient group.

The higher risk of late toxicity reported in the literature also led to concerns about the use of high doses in re-RT [7, 31, 32]. The current availability of modern technology with the possibility of high dose gradient techniques and image-guided delivery must encourage radiation oncologists to consider the new treatment options in the clinical setting of re-RT and safely deliver higher doses of 40–50 Gy with conventional fractionation. To date, new technologies allow us to use smaller margins, high dose gradient techniques and image-guided irradiation. This must give us the confidence to perform treatments that spares the organs at risk.

We have given to responders the possibility to choose between several options regarding the techniques used by the different centers. The quality of re-RT treatments was high, with modern conformal techniques (IMRT/VMAT) used in 96% of centers. Only a few centers considered the possibility of using 3DCRT techniques. All centers used protocols of daily IGRT (CBCT or MVCT).

The combination of highly conformal techniques and daily imaging protocols allowed the reduction of margins from the GTV to the CTV and from the CTV to the PTV. This, of course, also allows for low irradiation of OARs.

In addition, the time interval between initial treatment and re-treatment RT could also be correlated with the risk of toxicity. Tao et al. described that a shorter re-treatment interval of ≤ 24 months was associated with a higher rate of late toxicity [32]. In our survey, the appropriate time interval for re-RT was reported as at least 6 months in 53% of centers and 12 months in 44%. Only 3% of centers did not consider the time interval between the first treatment and re-RT.

One of the major and current weaknesses in the field of re-RT is that no OARs dose constraints are available for assessing the toxicity risk of re-RT in clinical practice. In our survey, we also investigated the maximum total dose considered in planning optimization for OARs, but only 4 radiation oncologists answered this question. The fact that so few radiation oncologists responded to this question is concerning and explains how little is known about re-RT for LR of rectal cancer. The low indication rate for re-RT may be due to fear of a topic that is ignored and difficult to study given the lack of available work. There is only one retrospective study in the literature that clearly stated the maximum cumulative dose to the bowel, rectum, and bladder for pelvic SBRT re-irradiation [25]. We need prospective studies to extrapolate dose constraints that can be recommended in clinical practice.

The survey certainly has its limitations. Sampling and non-response biases could be present, due to the response rate of 20% of the total radiotherapy centers in Italy and the geographical distribution of the responses, since only 11% of the responses came from regions in the south of Italy. This could be due to the fact that not all centers deal with re-irradiation of recurrent rectal cancer. Order bias could also be present, with an influence of the format employed on the chance to provide a specific response. The study relied on self-reporting, known to be potentially misaligned with reality and leading to potential recall and response biases. Nevertheless, a positive degree of concordance concerning high quality of re-RT treatments, with modern conformal techniques, was reported.

The increasing use of new technologies can have a further positive impact in this scenario.

The use of MR-LINAC, which not only allows visualization of the target throughout the duration of irradiation but also adapts the treatment to the anatomy of the day, taking into account the possible movement of the target and organs at risk, could allow the reduction of the CTV–PTV margin that could lead to irradiation with higher doses [33].

Carbon ions RT (CIRT) could also be a promising treatment option. Heavy ion beams could be beneficial both because of the physical selectivity of the particles with the high dose gradient and the efficacy of their radiation biology, with RBE reported to be 2–4 times higher than photons [34].

Finally, data from total neoadjuvant therapy (TNT) studies in locally advanced rectal cancer management may also influence the treatment of LR rectal cancer patients. In particular, the recent 5-year follow-up data of RAPIDO trial results showed that, although TNT is able to reduce the rate of distant metastasis, the rates of both locoregional failure and locoregional recurrence (LRR) were statistically higher in patients who underwent short-course radiotherapy followed by systemic chemotherapy and surgery than in patients who underwent chemoradiotherapy followed by surgery. Interestingly, however, the LRR rate in patients treated with 3D-RT was higher in the arm that received short-course radiotherapy followed by surgery than in the arm that received chemoradiotherapy followed by surgery, whereas the outcomes of IMRT/VMAT treatments of the two arms were comparable [35]. Since LR rectal cancer patients are likely to develop distant metastasis, intensive chemoradiotherapy regimens should also be considered as part of TNT, considering the new technologies and therapeutic scenario.

## Conclusion

Our survey showed that re-RT treatment is performed with advanced technology that allow a good management of rectal cancer LR in Italy.

Re-RT should be proposed in multidisciplinary discussion when evaluating a patient with recurrent rectal cancer, as it may lead to a reasonable chance of resectability and a better prognosis for the patient.

It is important to standardize classification criteria for recurrences, as this can lead to personalization of treatment. In addition, the radiotherapy currently offered in cases of recurrence is more precise and target-compliant, with a lower toxicity profile. To date, radiation treatment schedules of re-RT are based on dated published work.

Considering new technologies, as IGRT protocols, higher doses, as well as the integration with TNT strategies, can certainly be considered to improve local control.

The considerable variability reported in this survey underlies the need for a consensus on an integrated, multidisciplinary treatment strategy and for a prospective study that could validate new treatment schedules in terms of toxicity profile and therapeutic efficacy in recurrence rectal cancer.
